# Supplementary material for: Availability and geographic access to breast cancer pathology services in Ghana
Source: PLoS One. 2024 Aug 14;19(8):e0305901. doi: 10.1371/journal.pone.0305901 (PMC11324111; doi:10.1371/journal.pone.0305901)
Supplement: Attachment — Submitted filename: Response to Data Access Timeframe Query.docx [file pone.0305901.s001.docx]

**Matthew Price, MD MPH**

Center for Global Surgery

4 North 30 N. Mario Capecchi Dr.

Salt Lake City, UT 84112

13 February 2023

Dear Reviewer,

Thank you for bringing to light this issue. We believe we have appropriately responded to the issue raised. If there are further issues, please let us know.

1. Thank you for submitting your work to PLOS ONE. Before we can proceed, please report in the Methods section the dates when data were accessed for research purposes. Please note that if this information is not included when your manuscript is resubmitted, it may be rejected.
   a. The first author of this project, Matthew Price, accessed the data between January 1^st^ and March 1^st^ 2023 for the initial analysis and again between October 1^st^ and December 31^st^ 2023 for confirmatory analysis prior to manuscript submission. A formal data use agreement was signed on November 17^th^ 2022 between Dr. Sutherland at Ensign Global College and Matthew Price at Johns Hopkins. Matthew Price received formal IRB determination on November 28, 2022 that this work is not considered human subjects research as it pertains to secondary data analysis involving the use of existing data. Both the DUA and Johns Hopkins IRB determination form are attached to this submission.
   b. The following line has also been added to the methods section of the submission:
      i. The dataset for this project was accessed between January 1^st^ and March 1^st^ 2023 and again between October 1^st^ and December 31^st^ 2023 for confirmatory analysis.

Sincerely,

Matthew Price

Matthew Price, MD MPH
